# Siderophore Production by Rhizosphere Biological Control Bacteria *Brevibacillus brevis* GZDF3 of *Pinellia ternata* and Its Antifungal Effects on *Candida albicans*

**DOI:** 10.4014/jmb.1910.10066

**Published:** 2020-02-21

**Authors:** Miaomiao Sheng, Huake Jia, Gongyou Zhang, Lina Zeng, Tingting Zhang, Yaohang Long, Jing Lan, Zuquan Hu, Zhu Zeng, Bing Wang, Hongmei Liu

**Affiliations:** 1 Engineering Research Center of Medical Biotechnology, Guizhou Medical University, Guiyang 550025, Guizhou, P.R. China; 2 Immune Cells and Antibody Engineering Research Center of Guizhou Province, Key Laboratory of Biology and Medical Engineering, School of Biology and Engineering, Guizhou Medical University, Guiyang 55005, Guizhou, P.R. China; 3 School of Basic Medical Sciences, Guizhou Medical University, Guiyang 550025, Guizhou, P.R. China

**Keywords:** *Brevibacillus brevis* GZDF3, siderophore, *Candida albicans*, *Pinellia ternata*

## Abstract

*Brevibacillus brevis* GZDF3 is a gram-positive, plant growth-promoting rhizosphere bacterium (PGPR) isolated from the rhizosphere soil of *Pinellia ternata* (an important herb in traditional Chinese medicine). The GZDF3 strain produces certain active compounds, such as siderophores, which are the final metabolite products of non-ribosomal peptide synthetase (NRPS) and independent non-ribosomal peptide synthetase (NIS) activity. With the present study, we attempted to investigate the siderophore production characteristics and conditions of *Bacillus* sp. GZDF3. The antibacterial activity of the siderophores on pathogenic fungi was also investigated. Optimal conditions for the synthesis of siderophores were determined by single factor method, using sucrose 15 g/l, asparagine 2 g/l, 32°C, and 48 h. The optimized sucrose asparagine medium significantly increased the production of siderophores, from 27.09% to 54.99%. Moreover, the effects of different kinds of metal ions on siderophore production were explored here. We found that Fe^3+^ and Cu^2+^ significantly inhibited the synthesis of siderophores. The preliminary separation and purification of siderophores by immobilized-metal affinity chromatography (IMAC) provides strong antibacterial activity against *Candida albicans*. The synergistic effect of siderophores and amphotericin B was also demonstrated. Our results have shown that the GZDF3 strain could produce a large amount of siderophores with strong antagonistic activity, which is helpful in the development of new biological control agents.

## Introduction

Iron is essential for the growth and survival of most microorganisms. However, in the natural environment, iron is mainly present in the form of insoluble Fe^3+^ (10^-18^ M at pH 7.0). Therefore, it is difficult to obtain available iron from the surroundings for microorganisms [1, 2]. One of the most common strategies developed by bacteria, fungi and plants is the production of siderophores (from Greek, meaning “iron carriers”). Siderophores are natural iron chelators, with low molecular weight secondary metabolites (400–2,000 Da), and high affinity for Fe^3+^. They are secreted to acquire iron as needed by microorganisms [3-5]. So far, more than 500 kinds of siderophores have been reported, and these can be divided into hydroxamaces, catecholates, carboxylates and various other types according to the characteristics of their coordination structure [6]. In iron deficient environments, microorganisms secrete synthetic siderophores to obtain iron and maintain their growth. Some plant growth-promoting rhizobacteria (PGPR) can synthesize siderophores, compete with pathogens to obtain iron, and inhibit the growth of pathogenic microbes [7, 8].

We previously reported that *Brevibacillus brevis* GZDF3 (CGMCC No. 10121) was isolated from the rhizosphere soil of *Pinellia ternata*. It was a new biocontrol bacterium found via hot screen method isolated from the rhizosphere soil of *P. ternata* in Guizhou Province, China. *B. brevis* GZDF3 is used not only for control of pathogenic organisms such as *Pectobacterium carotovorum subsp. carotovorum*, but also the pathogenic fungi *Fusarium solani* and *Fusarium oxysporum*. The GZDF3 strain has strong antagonistic activity against other pathogenic microbes as well. Therefore, it has great potential for biocontrol [9]. In recent years, siderophores have drawn much attention due to their potent roles in the medical industry, agriculture, and environmental science [10]. For instance, siderophore enterobactin of *Escherichia coli* promotes mitochondrial iron uptake and the development of *Caenorhabditis elegans* via interaction with its ATP synthase [11]. Our previous research reported that *B. brevis* GZDF3 can produce catechol-type siderophores and has a strong antagonistic effect on *F. oxysporum*. However, factors that affect the production of siderophores and the antifungal effect against *Candida albicans* of *B. brevis* GZDF3 have not yet been reported [12].

Diverse factors, such as carbon source, nitrogen source, pH, temperature and metal ions have been reported to affect the synthesis of siderophores [13]. Moreover, maximum siderophore production conditions vary according to strain type [13, 14]. Bendale reported that maximum siderophore production could be achieved when sucrose was used as a carbon source by *Streptomyces fulvissimus*. Whereas in Santos’s study, glycerol was proven to be the favorite carbon source for the growth of *Bacillus megatherium* [13, 14]. Maximum siderophore production of *Bacillus* sp. PZ-1 was observed in the glucose concentration of 21.84 g/l [15]. The pH is also a crucial factor in siderophore production. Generally, a neutral pH is favorable for microorganisms to produce the maximum amount of siderophores in *fluorescent pseudomonads* [16]. In *Pseudomonas syringae* strain BAF.1, maximum siderophore production was achieved with glucose as a carbon source and asparagine as a nitrogen source, at 30°C and pH 7.0 [17].

It is important to note that many metal ions have shown the capacity to affect siderophore production [18, 19]. It was reported that Zn ion increased the siderophore production whereas Cu ion decreased it [20]. Addition of Cd also enhanced the production of siderophore in iron-limited cultures [19]. In fungal strains, the maximum siderophore production was detected at Zn ion concentration of 150 μg/ml [20, 21]. Moreover, the Pb ion could also stimulate PZ-1 strain siderophore production [15, 22].

*C. albicans* is an important opportunistic pathogen that can cause a variety of human infections, and the current treatment involves the use of antifungal agents, such as polyenes and azoles [23]. Ergosterol is a neutral lipid in fungal membranes, and being critical for many cell processes [24], the destruction of its synthesis has become the focus of antifungal therapy. Both types of antifungal agents cause the disorder of plasma membrane structure and function by combining ergosterol (polyenes, *e.g.*, amphotericin B) or inhibiting the enzyme lanosterol 14α-demethylase, which is involved in ergosterol biosynthesis (azoles, *e.g.*, fluconazole) [25]. Iron is an essential nutrient for all living cells. There is increasing evidence that interfering with the iron homeostasis of *C. albicans* can improve its antifungal response. It has been shown that iron-chelating agents can improve the activity of azole. The iron carrier, as an iron-chelating agent, is a potential new antibiotic and bacteriostatic agent [26]. Kimley has reported, that the new iron-chelating agent (DIBI) has stronger growth inhibition effect with various azole combinations than that of albicides alone. It greatly prolongs the inhibition of *C. albicans* cell proliferation [27]. In this study, we optimized the composition of *B.brevis* *GZDF3* medium and related culture conditions and investigated the effect of metal ions on the synthesis of iron carrier. The antibacterial activity of iron carrier against *C. albicans* was preliminarily verified, and the synergistic effect of siderophores with amphotericin B was demonstrated. Herein, these results provide new possibility for further understanding the mechanism of action of GZDF3 on *C. albicans* and its application in the cultivation of *Pinellia ternata*, while also serving as a useful reference for fighting fungal infection.

## Materials and Methods

### Microorganism Strains and Medium

*B. brevis* GZDF3 and *C. albicans* are preserved in the Department of Biotechnology, College of Biology and Engineering, Guizhou Medical University. The strain was subsequently grown on Luria-Bertani (LB) medium (10 g/l tryptone, 5 g/l yeast extract, 10 g/l NaCl) or Potato Dextrose Agar (PDA) medium (200 g/l potato, 20 g/l glucose). The bacterium was maintained in glycerol and kept at -80°C. To screen for siderophore production, we chose the optimized fermentation sucrose-asparagine (SA) medium (20 g/l sucrose, 2 g/l L-asparagine anhydrous, 1g/l KH_2_PO_4_, and 1 g/l MnSO_4_·7H_2_O).

### Chrome Azurol S (CAS) Liquid Detection

The strain GZDF3 was activated and the bacterial culture solution was prepared. The bacterial culture solution was added into 150 ml of SA medium at a dose of 5%, and cultured at 28°C and 200 rpm for 36 h, followed by centrifugation at 12,000 ×*g* for 10 min. The supernatant was removed by filtration using a 0.22 μm microporous membrane. Next, we mixed 1 ml of fermentation broth with an equal volume of CAS blue detection solution. The siderophore production was determined by CAS liquid test. The SU was calculated by the following formula: SU = [(Ar-As)/Ar]×100%. Where Ar represents the absorbance of reference (uninoculated broth), and As is the absorbance of the sample (culture supernatant) at 630 nm [28].

### SA-CAS Plate Assay

The universal CAS assay (Schwyn and Neilands 1987) was modified to test the production ability of microorganisms with siderophore-type, iron-binding compounds in solid medium avoiding growth inhibition caused by the toxicity of CAS-blue agar medium. CAS-blue agar (100 ml) was prepared according to Schwyn and Neilands (1987). Petri dishes (10 cm in diameter) were prepared with 30 ml of appropriate medium for culturing each strain. After solidifying, the medium was cut into halves, one of which was replaced by CAS-blue agar (15 ml). The halves containing culture medium (SA) were inoculated with strains taken from stock cultures. The inoculum was placed as far as possible from the borderline between the two media. Then, the plate was incubated for 48 h at 28°C. The CAS reaction was determined by measuring the position or distance of the advancing color-change front in the CAS-blue agar, starting from the borderline between the two halves during all incubation times [28].

### Optimization of the Cultural Conditions for Siderophore Production

A single-factor experiment was adopted to evaluate the effect of physicochemical parameters (fermentation time, culture pH, fermentation temperature, carbon source, nitrogen source and metal ion) on cell growth and siderophore production of GZDF3 strain. Cell growth was measured at 600 nm using a spectrophotometer [28]. The siderophore production was determined by CAS liquid test. The cell growth and siderophore production were measured as a function of fermentation time at 0, 6, 12, 18, 24, 30, 36, 42, 48, 54, and 60 h. The pH was adjusted to 4.0, 5.0, 6.0, 7.0, 8.0, 9.0, and 10.0, respectively. Fermentation temperature was set from 23°C to 37°C. Fructose, maltose, glucose, mannose, glycerol and xylose were used at a concentration of 20.0 g/l, replacing sucrose as a carbon source in SA medium. In addition, glutamic acid, lysine, tyrosine, tryptophan, and proline were used at a concentration of 2.0 g/l, substituting for asparagine as a nitrogen source in SA medium. The effect of metal ion was tested by supplementing SA medium with FeCl_3_•6H_2_O, CuSO_4_, MnSO_4_, and ZnSO_4_•7H_2_O at a concentration of 0, 5, 10, 20, 50, 100, 200, 400, and 800 μmol/l, respectively. All culture flasks were incubated in a shaker at 200 rpm and 28°C for 48 h, except for temperature experiments. The siderophore production was then quantified according to the above method.

### Preliminary Purification of Siderophore

After culturing for 48 h at 30°C and 200 rpm, the liquid culture was centrifuged at 1,000 ×*g* for 10 min and the culture filtrate was harvested. Then, the culture filtrate was mixed with 4 volumes of ethanol, precipitated overnight, concentrated by a rotary vacuum evaporator, resuspended in deionized water, and subjected to further study. The filtrate was used as the crude fermentation siderophore solution. The crude solution was then purified by Fe(III)-IMAC column (HiTrap Chelating HP/1 ml). The purified siderophore solutions were used in the antifungal experiments [29].

### Antagonistic Activity Analysis of the Siderophore Solution 

In this study, the punch method (agar cup method) was used to investigate the antifungal activity of the siderophore solution [30]. SA medium was inoculated with GZDF3, followed by incubation at 32°C for 48 h. Next, crude and purified siderophore solutions were obtained as described above.

PDA plates were prepared and used for antimicrobial screening. Overnight broth cultures of the test organisms were seeded on plates. After that, wells approximately 8 mm in diameter and 2 mm deep were made on the surface of the solid medium using a sterile borer. Each well was subsequently filled with 100 μl of test sample and labeled with a marker. Sterile SA medium was used as negative control, while ketoconazole was used as positive control. The test samples were 1 mg/ml, 5 mg/ml, 10 mg/ml of ketoconazole, purified siderophore, Fe^3+^-treated crude fermentation medium, GZDF3 solution fermented in SA medium for 36 h and GZDF3 solution fermented in the optimized SA medium for 48 h. All plates were incubated at 28°C for 36 h. Then, a photo was taken and analyzed. The zones of inhibition on the plate were measured (mm) by cross measurement method to evaluate the antifungal activity.

The inhibitory effects of different concentrations of siderophore on *C. albicans* at different times were measured by microplate reader, and time-kill curves were drawn out. *C. albicans* was cultured in the potato dextrose (PDB) liquid medium. Different concentrations of siderophore (2,500, 1,250, 625, 312.5, 156.3, 78.1, 39.1, 19.5 μg/ml) were purified by Fe (III)-IMAC column and added to the medium. The PDB medium-only group (Medium) was used as a negative control. *C. albicans* with PDB medium (Bacteria Solution) was used as a control group. Amphotericin B (19.5 μg/ml) (AMP) was used as a positive control. Then, samples were detected by microplate reader at different time points (0, 2, 4, 6, 8, 10, 12, 14, 16, 18, 20, 22, 24, 26 h). The OD_600_ value represents the bacteria concentration of *C. albicans*. Data are representative of three independent experiments.

### Checkerboard Assay

The checkerboard assay for siderophores and amphotericin B was performed on the 96-well microtiter plate with the concentration of siderophore ranging from 2,040 μg/ml to 0 μg/ml, and the concentration of amphotericin B ranging from 200 μg/ml to 0 μg/ml, multiple proportion dilution. Each well contained the starting inoculum of approximately 1.5×10^8^ CFU/ml, and the predefined concentration of each drug in the total volume of 50 μl. The 96-well plate was oscillated at 28°C for 150 rpm/min for 24 h, the turbidity of the medium was observed, and the absorbance of OD_492_ nm was measured. Each combination was performed in triplicate. The fractional inhibitory concentration index (FICI) for the two drugs was calculated as follows:

$$\mathrm{FICI}=\frac{\mathrm{MIC}(\mathrm{siderophore})}{\mathrm{MIC}(\mathrm{siderophore})\mathrm{com}}+\frac{\mathrm{MIC}(\mathrm{amphotericin}A)\mathrm{com}}{\mathrm{MIC}(\mathrm{amphotericin}A)\mathrm{com}}$$

The interaction between the two drugs was interpreted as synergistic if the FICI was ≤0.5, indifferent if it was > 0.5 and ≤ 4, and antagonistic if it was > 4 [31].

### Statistical Analysis

Data were analyzed using SPSS 19.0 (SPSS Inc., USA) and expressed as the mean ± standard error (SE). Statistical analyses were carried out using one-way analysis of variance. The statistical significance of the test was evaluated at the level of *p* < 0.05 regarded as reliability.

## Results

### Detection of Ferritin Synthesis by CAS Liquid and Plate Method

**Fig. 1:**
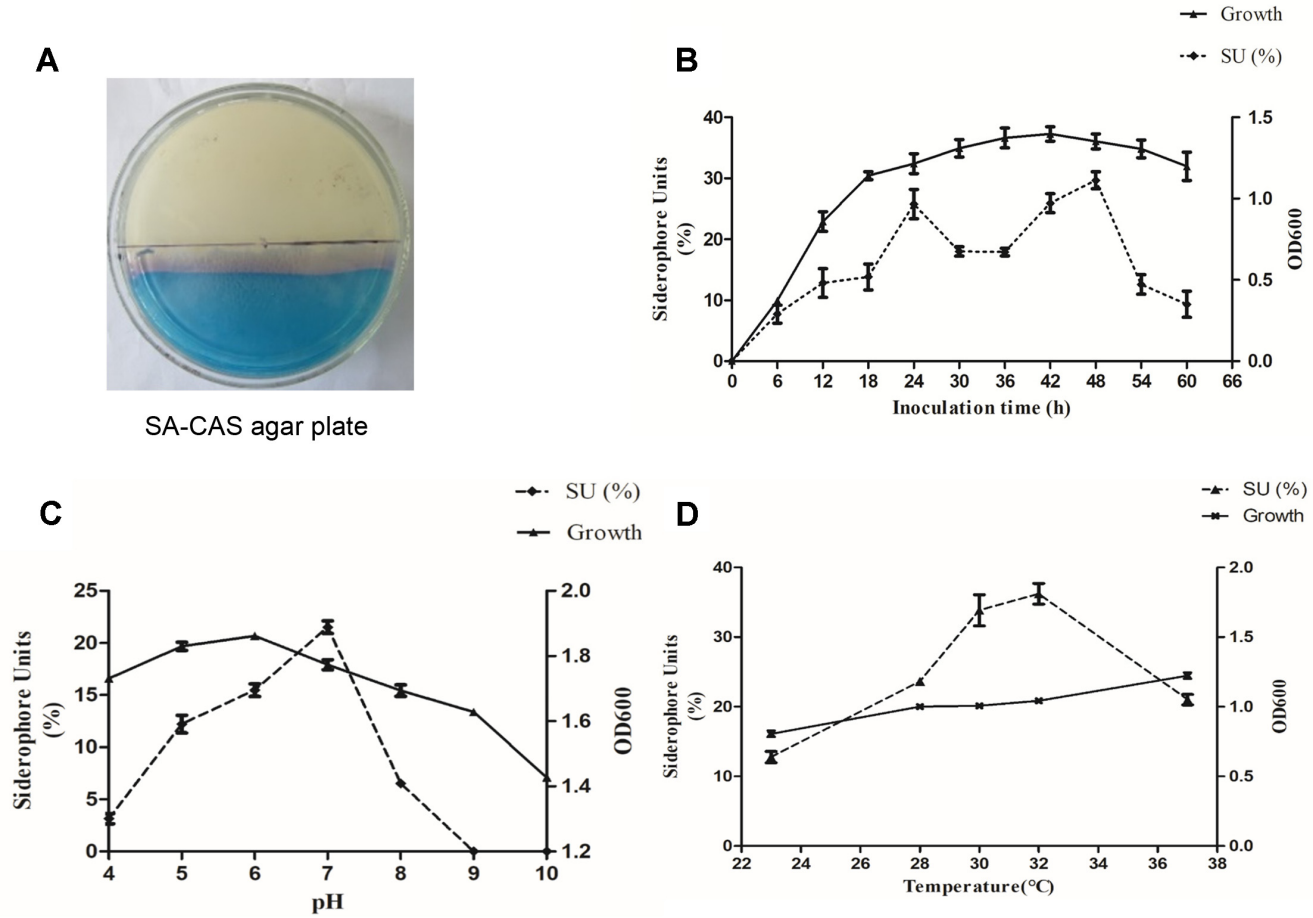
Effect of fermentation time, pH, temperature on siderophore production and cell growth of GZDF3. (**A**) GZDF3 was cultured on SA-CAS agar plate, and the pink region means GZDF3 produces the siderophore. (**B**) The siderophore and cell growth curve graph. GZDF3 was cultured in SA medium and collected sample at different time points as shown in figure. OD600 was detected via multiscan spectrum instrument to evaluate the cell growth (right vertical axis). Siderophore content was determined by CAS liquid assay as described in Materials and Methods (left vertical axis). (**C, D**) Similar to (**B**), GZDF3 was cultured in SA medium with different pH (**C**), temperature (**D**) as shown in figure. And sample was collected and detected as described above. All data were collected and analyzed in one graph. Error bars represent standard deviations of three replicates.

After culturing *B. brevis* GZDF3 for 36 h in SA medium, Fe^3+^ in CAS-Fe^3+^-HTDMA was transferred to iron-facilitated as the ability of siderophores to chelate iron ions stronger than that of CAS-Fe^3+^-HTDMA complex. As shown in Fig. 1A, a pale pink color change has appeared on the CAS blue solid agar of the SA-CAS plate. It has been demonstrated that the GZDF3 strain produced siderophores during growth on the surface (SA medium). The color change due to the production of siderophores was dynamically observed. The color reaction is consistent with the liquid detection, which further confirms that *B. brevis* GZDF3 can produce siderophores.

Secondly, different fermentation time points were chosen from 0 to 60 h with 6-h intervals to investigate their effects on siderophore production and cell growth. As shown in Fig. 1B, the cell growth and siderophore production varied greatly with different fermentation times. The growth curve of GZDF3 was not a typical bacterial growth curve. The lag phase encompassed the initial 6 h. In the logarithmic phase, from 6 h to 42 h, cells kept growing (Fig. 1B, upper line). Thereafter, it entered the decline phase. The siderophore production trend was different from that of cell growth. We found that siderophores could be detected after 6 h. In addition, the maximal production of siderophores was 29.67% measured at 48 h. It is noteworthy that the siderophore production of 25.76% reached another peak when the incubation time was 24 h (Fig. 1B, lower line). Thus, 48 h was chosen as the best fermentation time in the following experiments (Table 1).

**pH of Medium.** As shown in Fig. 1C, the siderophore production was influenced more obviously than the growth of GZDF3 by pH factor. The optimum pH for cell growth was 6.0. However, the highest siderophore production (21.27% SU) was obtained at pH 7.0. Therefore, the pH value was set to 7.0 in the following experiments for optimal siderophore production (Table 1).

**Fermentation temperature.** In our previous study, GZDF3 grew well at 37°C [19]. Taking soil temperature into account, the fermentation temperature was set from 23°C to 37°C. The result showed that fermentation temperature not only affects the bacterial growth but also the siderophore production. In Fig. 1D, the highest yield of siderophore (35.46% SU) occurred at 32°C. However, the siderophore production decreased quickly as temperature increased above 32°C. Interestingly, little change occurred in the GZDF3 cell growth as the temperature was increased. This indicated that fermentation temperature had little influence on cell growth. Hence, the temperature of 32°C was also used in the following experiments (Table 1).

### Effects of Media Composition on Siderophore Production and Cell Growth

*B. brevis* GZDF3, belonging to Bacillus, was isolated from the rhizosphere soil of *Pinellia ternata* in Da Fang County, Guizhou Province, China. It has potential for use as biocontrol bacteria to cure pathogenic bacterial infection using *P. ternata* (an important herb in traditional Chinese medicine). Due to different microorganisms preferring different types and concentrations of media and fermentation conditions (temperature, time, etc.), we attempted to optimize these growth conditions for *B. brevis* GZDF3. As described in previously published papers, the culture medium for siderophore production of *Bacillus* sp. PZ-1 is modified SA medium [15, 17]. Using published papers on SA medium as reference [32, 33], we chose SA medium as the basic medium to optimize the siderophore production and growth of *B. brevis* GZDF3.

**Fig. 2:**
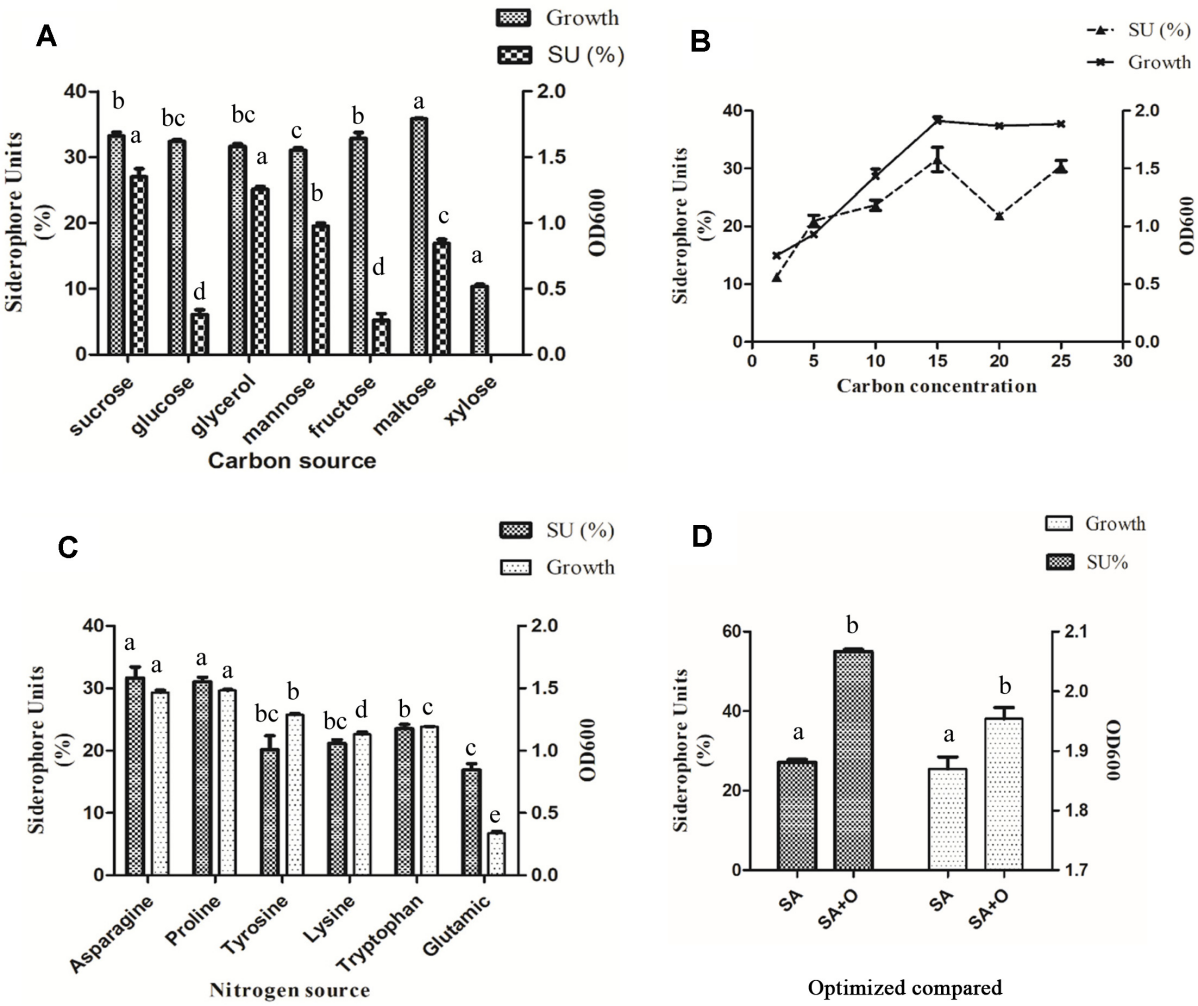
Effect of carbon, nitrogen source on siderophore production and cell growth, and compared to the control under optimized culture conditions. GZDF3 was cultured on SA medium, with different carbon source (sucrose, glucose, glycerol, mannose, fructose, maltose, xylose), SA+O (optimized SA medium); (**A**) or different nitrogen source (Asparagine, Proline, Tyosine, Lysine, Tryptophan, Glutamic) (**C**) as shown in figure. (**B**) GZDF3 was cultured in SA medium with different concentrations sucrose as carbon source. (**D**) Changes in siderophore production and cell growth before and after optimization. And samples were collected and detected as described above. OD_600_ represent bacteria concentration of GZDF3 (right vertical axis) and SU means siderophore units (left vertical axis). Bars with the same letter are not significantly different (*p* ≥ 0.05) Error bars represent standard deviations of three replicates.

In order to determine the optimal carbon source to induce higher siderophore production, carbon sources, including glycerol, monosaccharides (fructose, glucose, and mannose) and disaccharides (xylose, maltose, and sucrose) were tested. The cell growth and siderophore production were influenced by the carbon source. As shown in Fig. 2A, siderophore production varied greatly with carbon source, and the maximum production of siderophore (27.02 % SU) was obtained with sucrose. Glycerol ranked in second place, inducing high siderophore production (25.10 % SU), whereas glucose, mannose, fructose, and maltose were at 6.08%, 19.48%, 5.21%, and 16.87%, respectively. On the contrary, siderophore production couldn’t be detected with xylose as the carbon source. It is obvious that maltose is the most favorable carbon source for GZDF3 growth, whereas xylose is the most unfavorable in comparison with the other options.

The concentration of sucrose was optimized, as shown in Fig. 2B. It was observed that when the sucrose concentration was 15 g/l, the highest siderophore production reached 31.59%. Therefore, the following experiments were all performed with 15 g/l sucrose as the carbon source (Table 1).

As shown in Fig. 2C, various amino acids showed different effects on siderophore production. Asparagine induced the highest siderophore yield of 31.64%, and the minimum production of siderophore at 16.92% was obtained with the addition of glutamic acid. Therefore, asparagine was used as nitrogen source in the following experiments (Table 1). As shown in Fig. 2D and Table 1, the optimized SA medium (SA+O) significantly increased the production of siderophores, from 27.09% to 54.99%, and also promoted cell growth in comparison with the basic SA medium.

### Effects of Metal Ions on Siderophore Production and Cell Growth

Due to the influence of metal ions on siderophore production [19, 21], four metal ions (Fe^3+^, Mn^2+^, Cu^2+^, Zn^2+^) were investigated for their effects on siderophore synthesis in this study.

**Fig. 3:**
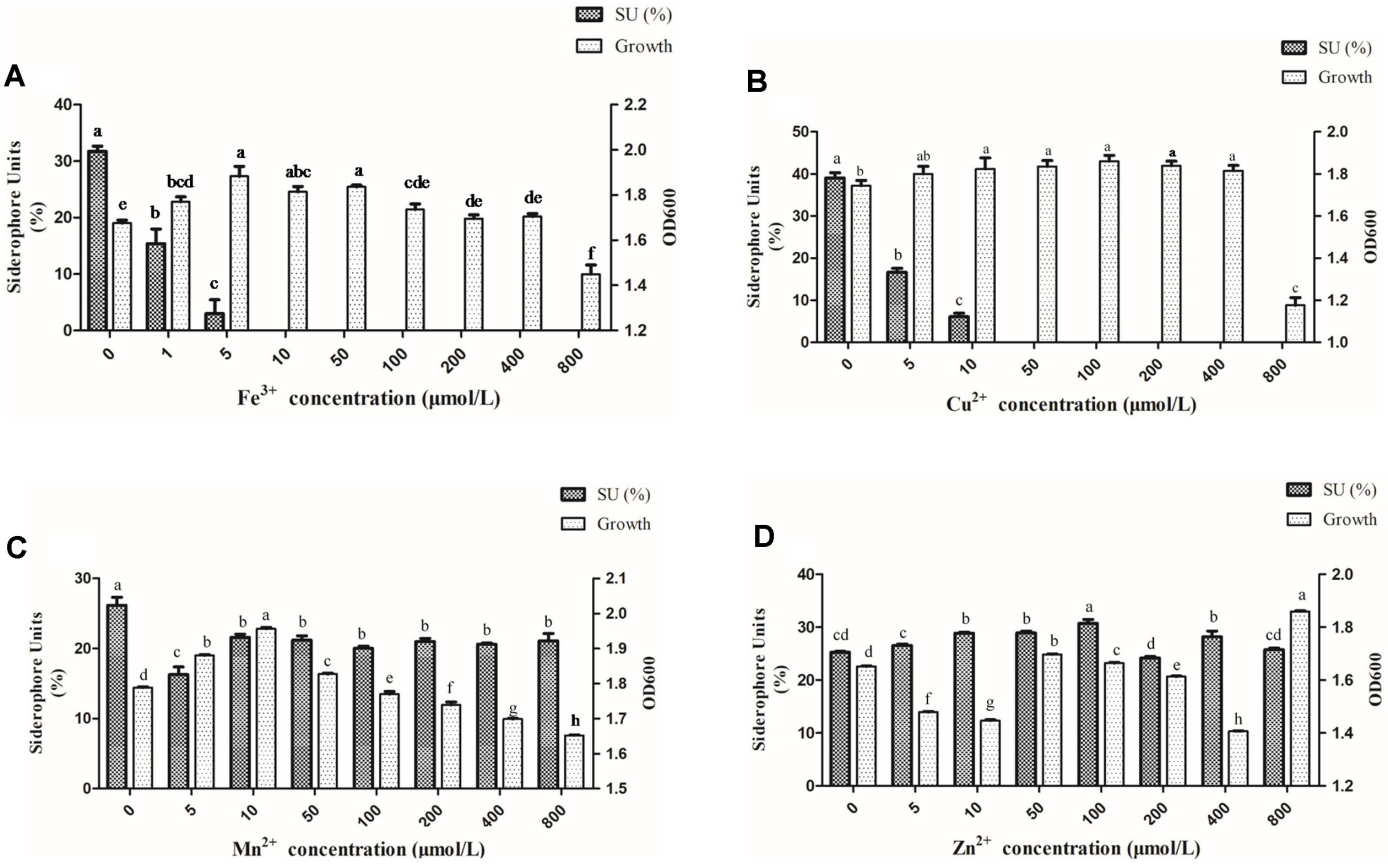
Effect of metal ions on siderophore and bacterial growth. Similar to Fig. 2, GZDF3 was cultured in SA medium with different concentrations of metal iron (Fe^3+^, Mn^2+^, Cu^2+^, Zn^2+^) as shown in figure. And sample was collected and detected as described above. OD600 represent bacteria concentration of GZDF3 (right vertical axis) and SU means siderophore units (left vertical axis). Error bars represent standard deviations of three replicates. Bars with the same letter are not significantly different (*p* ≥ 0.05) Error bars represent standard deviations of three replicates.

As shown in Fig. 3, different concentrations of ferric ion (0, 5, 10, 50, 100, 200, 400, and 800 μmol/l) of FeCl_3_ were added to the SA medium to assess their function on siderophore production. The increase of Fe^3+^ concentrations had a negative effect on siderophore production, especially when the concentration was above 10 μmol/l. A significant reduction trend of siderophore production was found as FeCl_3_ concentration was increased. It is interesting to note that the highest siderophore production was without Fe^3+^ medium. As FeCl_3_ concentration increased from 1 to 5 μmol/l, the amount of produced siderophore decreased by 80%. This production was almost completely suppressed when FeCl_3_ was at 10 μmol/l. Meanwhile, increased cell growth was observed as the FeCl_3_ concentration increased from 0 to 10 μmol/l. However, the cell growth was slightly repressed when FeCl_3_ concentration was higher than 10 μmol/l (Fig. 3A).

Similar to Fe ion, the siderophore production was also suppressed by the increased Cu^2+^ concentration. There was nearly no siderophore production at the concentration of 50 μmol/l (Fig. 3B). At the same time, no significant change was observed for the cell growth from 0 to 400 μmol/l. But the cell growth was decreased with 800 μmol/l of Cu^2+^ (Fig. 3B). This may because there is a tolerance range of GZDF3 growth to the concentration of Cu^2+^. If it is within a certain range, the growth won’t change too much. If the concentration of Cu^2+^ changes very much, this will be reflected in the growth of GZDF3.

The amount of siderophores decreased as the concentration of Mn^2+^ increased in this study, especially at the concentration of 5 μmol/l (Fig. 3C). Interestingly, the Mn^2+^ was observed to stimulate siderophore production and cell growth with the concentration from 5 to 50 μmol/l as shown in Fig. 3C. No significant change in the siderophore production was observed with Mn^2+^ concentration increasing from 100 to 800 μmol/l. Meanwhile, the cell growth was remarkably decreased (Fig. 3C).

As shown in Fig. 3D, it is interesting to find that Zn^2+^ had a relatively weak effect on siderophore production in comparison with the other metals. There was only a slight increase in siderophore production with increasing Zn^2+^. The cell growth was inhibited by increasing Zn^2+^ from 0 to 10 μmol/l. However, the inhibition effect did not show a clear trend with the increase of Zn^2+^, especially from 50 to 200 μmol/l.

These results indicated that the effect of metal ions on siderophore production of GZDF3 varied with metal species and concentration. It would not benefit siderphore production of GZDF3 to add metal ions (Fe^3+^, Cu^2+^, Mn^2+^, Zn^2+^) in the fermentation process.

### Antifungal Activity of Crude Siderophore Solution

As shown in Table 2, *C. albicans* growth was inhibited by GZDF3 fermentation solution which contained siderophore. The inhibition zones of GZDF3 fermentation broth with SA medium and optimized SA medium were 41 ± 3 mm and 45 ± 3 mm, respectively. In order to investigate the dose inhibition effect of siderophore to fungal growth, 1 mg/ml, 5 mg/ml, and 10 mg/ml Fe (III)-IMAC column-purified siderophore solutions were added to the hole in the plate. Ketoconazole at 1 mg/ml, 5 mg/ml, and 10 mg/ml was used as positive control, while 1 mg/ml, 5 mg/ml, and 10 mg/ml of Fe^3+^-treated fermented broth were used as complementary comparison experiment control. As showed in Table 2, both inhibition zones of purified siderophore and ketoconazole become wider as the dose was increased. On the other hand, only ketoconazole was observed with a small inhibition zone of 12 ± 3 mm. However, purified siderophore has a wider inhibition zone relative to ketoconazole. Interestingly, the Fe^3+^-treated fermentation medium showed decline of the inhibition zone. This is consistent with the inhibition effect of Fe (III)-IMAC column-purified siderophore to *C.albicans*. When we use the Fe (III)-IMAC column to purify siderophore, the siderophore concentration is higher in the solution. However, Fe^3+^ treatment inhibits the siderophore production in the fermentation medium. Thus, the inhibition effect to *C.albicans* was attenuated. According to the results in Table 1, strain GZDF3 is highly antagonistic to the pathogen *C. albicans*. Also, siderophore plays an important role in this effect.

**Fig. 4:**
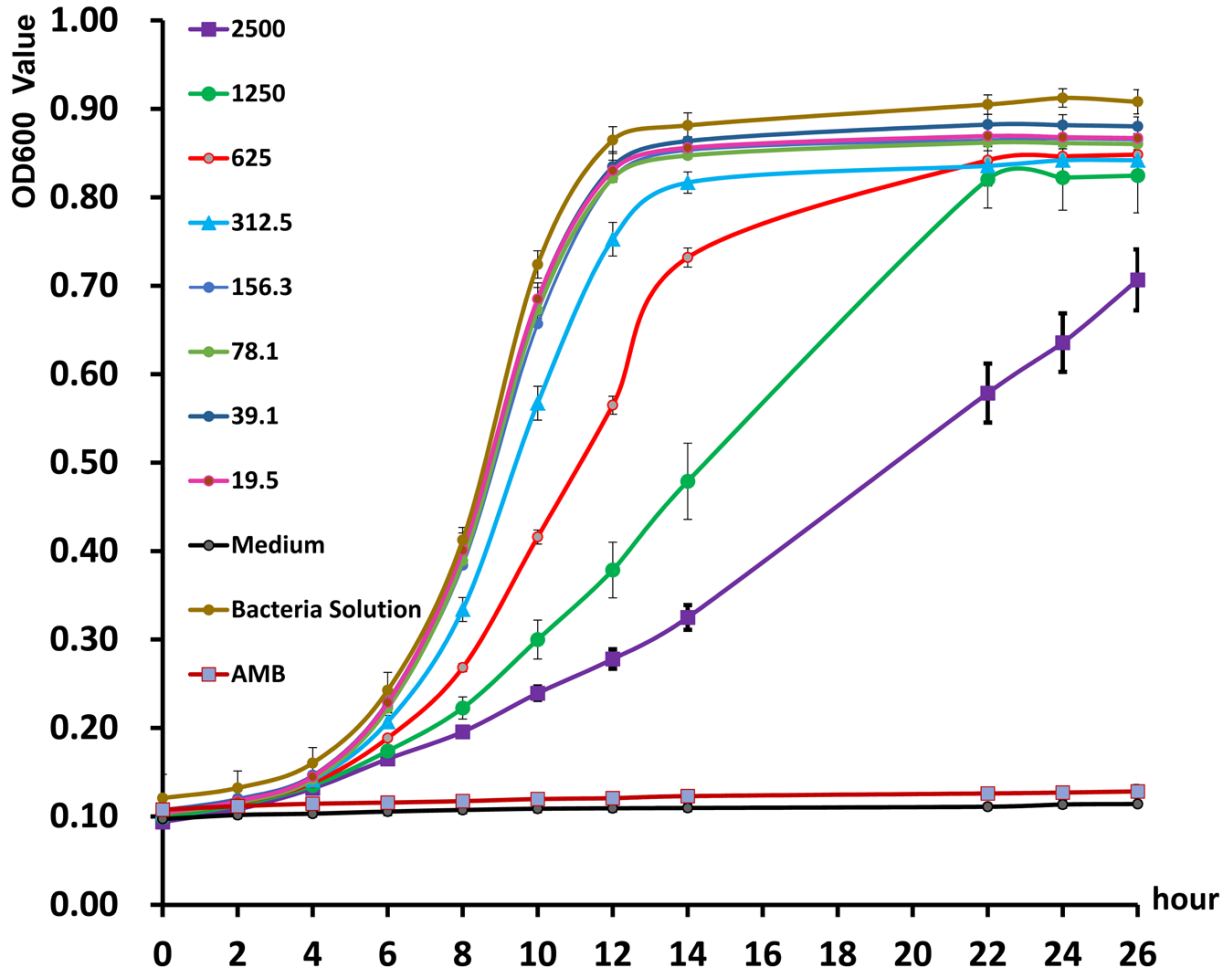
Time-kill curves of *Bacillus brevis* GZDF3 siderophore to *C. albicans*. *C. albicans* were cultured in the potato dextrose broth (PDB) medium. Different concentrations siderphore which was purified by Fe(III)-IMAC column were add to the medium according to the figure. PDB medium-only group (Medium) used as negative control. *C. albicans* with PDB medium (Bacteria Solution) is control group. And the amphotericin B (AMB) used as posititive control according to the figure. Then samples were detected by at different time points as described in the figure. OD600 value represents bacteria concentration of *C. albicans*. Error bars represent standard deviations of three replicates.

In order to further investigate the antifungal effect of GZDF3 siderophore to *C. albicans*, the time-kill curve was drawn with different concentrations of siderophores and different time points. Here, 2,500, 1,250, 625, and 312.5 μg/ml of purified siderophores all showed strong inhibitory effect to *C. albicans* throughout the whole period (Fig. 4). Moreover, the inhibition effect is decreased as the concentration reduces. Meanwhile, all of the four concentrations of siderophores have obvious inhibitory effect from 6 h to 12 h. These results show that the siderophores have significant effect during the 6th to 12th hour (Fig. 4) and also that purified siderophore has a significant antagonistic effect on *C. albicans*.

Siderophore produced by GZDF3 has a stronger antifungal effect than ketoconazole. Siderophore regulates intracellular ferrous ion for survival whereas ketoconazole and amphotericin B inhibit levels of ergosterol, which is one of the components in the membrane. The elucidation of relationship (synergistic or additive effect) between siderophore and antibiotics would be quite useful information. Due to ketoconazole having been reported for drug resistance phenomenon in clinical use, the synergistic or additive effect experiments were carried out with siderophore and amphotericin B in subsequent experiments.

As shown in Table 3, both siderophore and amphotericin B have been associated with great decline in MIC concentration when these two are combined together in comparison with being used alone. Also, the FIC value is 0.031, which is less than 0.5. It has been shown that siderophore produced by GZDF3 could have a synergistic effect on *C. albicans* when combined with amphotericin B.

## Discussion

In this study, we characterized the siderophore production of *B. brevis* GZDF3 (Fig. 1). The optimal fermentation conditions of siderophore production by *B. brevis* GZDF3 were reported as shown in Fig. 2 and Table 1. In addition, adding metal ions (Fe^3+^, Cu^2+^, Mn^2+^, Zn^2+^) in the fermentation process inhibited the siderophore production of GZDF3 (Fig. 3). Moreover, Fe(III)-IMAC column-purified siderophores of GZDF3 have obvious great anti-fungal activity to *C. albicans* (Fig. 4 and Table 2). In addition, GZDF3 siderophore possesses a synergistic resistance effect with amphotericin B to *C. albicans* (Table 3).

Culture medium and various abiotic factors could influence the siderophore production. The degree of acidity or alkalinity (pH) is a significant factor in siderophore production by *P. aeruginosa* PSS [34]. The maximum siderophore production was obtained in *Pseudomonas*, such as *Pseudomonas syringae* BAF.1, *Pseudomonas aeruginosa* and *fluorescent pseudomonads*, with medium of pH 7.0 [16, 17, 34]. However, pH 6.0 also proved suitable for producing hydroxamate siderophore in marine *Pseudomonas aeruginosa* and *Bacillus* sp. PZ-1 [15, 17, 35]. The maximum siderophore production (SU) of *B. brevis* GZDF3 was obtained at pH 7.0, which is similar to the conclusions drawn above. To sum up, a neutral pH is likely suitable for siderophore production of microbes, and our results show the same (Fig. 1 and Table 1).

Pyoverdine levels were much higher at 15°C to 20°C, but maximum cell growth was between 20°C and 30°C in *Pseudomonas fluorescens* [16]. In *Pseudomonas aeruginosa*, maximum siderophore production was recorded at 30°C. These were similar in GZDF3 for maximum siderophore yields at 30°C to 32°C in our study (Fig. 1B). It was reported that the siderophore production reached the peak when the fermentation time was 24 h for incubation of *Alcaligenes faecalis* BCCM ID 2374 [36, 37]. It was shown that the maximum siderophore production by *Bacillus megaterium* was obtained in the stationary phase [38]. Interestingly, the optimal fermentation time for siderophore production of GZDF3 has two peaks (24 h, 48 h) in our study. The first one is at 24 h and is weaker than the one at 48 h. Meanwhile, the cell growth of GZDF3 increases from 24 h to 48 h. Thus, inoculation of GZDF3 for 48 h in stationary phase is a good choice, all things considered.

Carbon source and nitrogen source determined the metabolism of microbes and also the quality of medium. Carbon sources such as sucrose and glycerol are important factors in cell growth and siderophore production of microbes. It is good for siderophore production in *Streptomyces fulvissimus* when sucrose is used as carbon source [13]. However, another report found that the highest siderophore production was detected with glycerols as carbon source in *Bacillus megaterium* [14]. Glucose was also found to influence the siderophore production in *P. fluorescens* CHA [39]. In *Pseudomonas syringae* BAF.1 and *Bacillus* sp. PZ-1, glucose was also the suitable carbon source to induce siderophore production [15, 17]. Herein, sucrose was proved to be the best carbon source to stimulate siderophore production in SA medium in our study (Fig. 2A).

Many reports found that culture media containing asparagine showed highly effective siderophore production by *Pseudomonas syringae* and *Pseudomonas viridiflava* LMG2352 strains [40]. We also found that asparagine and proline are suitable for inducing siderophore production and cell growth of GZDF3. However, the SU is higher in GZDF3 with asparagine as nitrogen source than proline. Thus, these results are mainly in accordance with those reported in *Pseudomonads* [35, 39, 40].

Metal ions have been reported to regulate siderophore production of microbes in the environment [41, 42]. In the present study, four metal ions (Fe^3+^, Cu^2+^, Mn^2+^, Zn^2+^) were selected to evaluate the effect on siderophore production of GZDF3. Surprisingly, the production of siderophore was suppressed by Fe^3+^ (Fig. 3). When the concentration of Fe ion reached to 10 μmol/L, siderophore could not be detected. This result was similar to that of *Pseudomonas syringae* BAF.1 [17]. However, it was found that the threshold level of Fe^3+^ suppressing siderophore production in gram-positive isolates was more than 30 μmol/l [16].

As shown in Fig. 3A, siderophore production is decreased in a Fe^3+^ concentration-dependent manner. The inhibition zone of 1 mg/ml Fe^3+^-treated GZDF3 fermentation medium is undetected. Also, as the concentration of Fe^3+^ increased to 5 mg/ml, the inhibition zone also increased to 19 ± 3 mm. However, the inhibition zone only changed to 20 ± 3 mm when the concentration of Fe^3+^ changed to 10 mg/ml. On the other hand, the inhibition zones of 10 mg/ml of purified siderophore or ketoconazole are 38 ± 3 or 30 ± 3 mm, respectively. These data show that the size of the inhibition zone is not increased in a typical Fe^3+^ concentration-dependent manner totally in Table 2. As Fe^3+^ concentration increased from 0 to 1 mg/ml, the siderophore production nearly decreased by half. So, the antifungal effect attenuated greatly, while the inhibition zone could not be detected at the same time. In addition, as the concentration of Fe^3+^ increased to 5 mg/ml, the inhibition zone appeared and didn’t change with further increase of the Fe^3+^. This indicated to us that there is probably some antifungal substance produced by GZDF3 with the stimulation of low-dose Fe^3+^(5 mg/ml). However, the substance didn’t increase as the Fe^3+^ concentration increased (10 mg/ml). Thus, the inhibition zone didn’t change much (from 19 to 20 mm). Also, as shown in Fig. 3, Table 2, the optimized SA medium and 10 mg/ml of purified siderophore have great antifungal effect with inhibition zones of 45 ± 3 or 38 ± 3 mm. These results showed siderophore is one of the key components of fermentation medium produced by GZDF3 and plays an important role in resistance to *C.albicans*.

It was reported that Al^3+^, Cd^2+^, Cu^2+^, and Ni^2+^ stimulated siderophore production by *Streptomyces* sp. [19]. One μM concentration of CuCl_2_, MgCl_2_, and ZnSO_4_ resulted in optimal siderophore yield in *Alcaligenes faecalis* [33, 34]. In contrast, we discovered that Cu^2+^ has the same effect on siderophore production as Fe^3+^. The higher the amount of Cu^2+^ added, the lower the amount of siderophore that was produced. The threshold level of Cu^2+^ that inhibited siderophore production in GZDF3 was above 50 μmol/l (Fig. 3).

It has been reported that the inhibition of siderophore synthesis in *zotobacter vinelandii* was found at elevated levels of Mn^2+^ and Zn^2+^ [37]. Surprisingly, in this study, Mn^2+^ was found to increase growth of GZDF3 with the concentration increased from 0 to 50 μmol/l, while the siderophore production decreased (Fig. 3C).

Reports show that high concentrations of Zn^2+^ ion increased the production of siderophore in iron-limited cultures in fungi and bacteria [20, 21]. However, no significant trend was found in siderophore production and cell growth by GZDF3 with the increase of Zn^2+^ in comparison with the other metals in our study (Fig. 3). These results suggested that the effect of metal on siderophore production varied with metal species and concentrations of GZDF3. Fe^3+^ and Cu^2+^ could strongly inhibit the siderophore production of GZDF3 as the increase in medium, and Mn^2+^, Zn^2+^ have weaker effects (Fig. 3).

Siderophore produced by *Alcaligenes feacalis* showed suppressive activity against *F. oxysporum* NCIM1008 [36]. Many reports suggested that siderophores produced by *P. fluorescens* MPF47 have strong biocontrol abilities against *R. solani* [43]. Moreover, it was also discovered that siderophore produced by *Seudomonas syringae* BAF.1 exhibited prominent antagonistic activity against *Fusarium oxysporum* in the absence of FeCl_3_•6H_2_O [17]. Interestingly, siderophore named *Bacillibactin* produced by SQR9 was up-regulated when it was confronted with fungi [44]. It was also shown that siderophores produced by *Pseudomonas fluorescens* BBc6R8 inhibited the growth of the actinomycete *Streptomyces ambofaciens* ATCC23877 [45, 46]. Compared with purified siderophores, cell-free supernatant of *P. putida* had a greater antagonistic activity towards fungi [47]. All of this reminds us that siderophores have anti-fungal activity [45, 48]. Yasmin reported the importance of siderophore and ergosterol biosynthetic pathways for fungal virulence and antifungal treatment, as iron starvation down-regulates the cellular ergosterol content but up-regulates siderophore (TAFC) production by *Aspergillus fumigatus* [26]. In our research, both crude and Fe(III)-IMAC column-purified siderophore solution produced by GZDF3 showed prominent antagonistic activity to *C. albicans* (Fig. 4 and Table 2). This should be useful in the prevention and control of fungus pathogens.

Siderophore regulates intracellular ferrous ion for survival, whereas ketoconazole inhibits ergosterol, which is one of the components in the membrane. The action mode of amphotericin B leads to pore formation on the membrane by binding to ergosterol, thereby leaking intracellular essential components. The elucidation of the relationship (synergistic or additive effect) is interesting. Iron is an essential nutrient for all living cells. There is increasing evidence that interfering with the iron homeostasis of *C. albicans* can improve its antifungal response. In line with this, our results show that siderophore produced by GZDF3 could act synergistically with amphotericin B when used against *C. albicans*.

In conclusion, we optimized the fermentation conditions of siderophore production by GZDF3, and characterized the siderophore’s antifungal function against *C. albicans*. These results could provide a useful reference for understanding the antifungal activity of GZDF3 to other fungal pathogens and its potential application in the cultivation of *Pinellia ternata*. However, further study is required to explore the exact antagonistic mechanism of siderophore produced by GZDF3 to *C. albicans* in the future.

## Figures and Tables

**Table 1:** Optimum conditions for synthesis of siderophore.

	Carbon	Nitrogen	Temperature	Time	pH	SU
**SA pre-optimized**	Sucrose 20g/l	Asparagine 2g/l	28°C	36 h	pH=7.6	**27.09%**
**SA optimized**	Sucrose 15g/l	Asparagine 2g/l	32℃	48 h	pH=7.0	**54.99%**

**Table 2:** Antifungal activity of GZDF3 against *Candida albicans*.

*Candida albicans*	Zone of inhibition (mm)
SA medium pre-optimized	41±3
SA medium optimized	45±3
1mg/ml ketoconazole	12±3
1 mg/ml purified siderophore	(—)
1 mg/ml Fe^3+^ medium	(—)
5 mg/ml ketoconazole	20±3
5 mg/ml purified siderophore	30±3
5 mg/ml Fe^3+^ medium	19±3
10 mg/ml ketoconazole	30±3
10 mg/ml purified siderophore	38±3
10 mg/ml Fe^3+^ medium	20±3

**Table 3:** Synergistic MIC value and FIC index of each drug when combined.

	MIC		FIC

	Combine	Alone	
Siderophore	64	1020	0.031
Amphotericin B	0.781	19.5	

Note: FIC index <0.5, Synergistic effect
